# Kinematic changes of the trunk and lower limbs during voluntary lateral sway postural control in adults with low back pain

**DOI:** 10.3389/fbioe.2024.1351913

**Published:** 2024-02-27

**Authors:** Zhengquan Chen, Oren Tirosh, Jia Han, Roger Adams, Doa El-Ansary, Adrian Pranata

**Affiliations:** ^1^ Shanghai Yangpu District Mental Health Center, Shanghai University of Medicine and Health Sciences, Shanghai, China; ^2^ Department of Nursing and Allied Health, School of Health Sciences, Swinburne University of Technology, Hawthorn, VIC, Australia; ^3^ College of Rehabilitation Sciences, Shanghai University of Medicine and Health Sciences, Shanghai, China; ^4^ School of Health and Biomedical Sciences, RMIT University, Melbourne, VIC, Australia; ^5^ Research Institute for Sport and Exercise, University of Canberra, Canberra, ACT, Australia; ^6^ Department of Surgery, Melbourne Medical School, Melbourne, VIC, Australia

**Keywords:** postural control, low back pain, biomechanics, gait, proprioception

## Abstract

**Introduction:** Voluntary lateral weight shifting is essential for gait initiation. However, kinematic changes during voluntary lateral weight shifting remain unknown in people with low back pain (LBP). This study aims to explore the differences in kinematics and muscle activation when performing a voluntary lateral weight shifting task between patients with LBP and asymptomatic controls without pain.

**Methods:** Twenty-eight participants volunteered in this study (14 in both the LBP group and the control group). The Sway Discrimination Apparatus (SwayDA) was used to generate a postural sway control task, mimicking lateral weight shifting movements when initiating gait. Kinematic parameters, including range of motion (ROM) and standard deviation of ROM (Std-ROM) of the lumbar spine, pelvis, and lower limb joints, were recorded using a motion capture system during lateral weight shifting. The electroactivity of the trunk and lower limb muscles was measured through surface electromyography using root mean square (RMS). The significant level was 0.05. An independent *t*-test was employed to compare kinematic parameters, and muscle activation between the LBP group and the control group. A paired-sample *t*-test, adjusted with Bonferroni correction (significant level of 0.025), was utilized to examine differences between the ipsilateral weight shifting towards side (dominant side) and the contralateral side.

**Results:** The results of kinematic parameters showed significantly decreased ROM and std-ROM of the ipsilateral hip in the transverse plane (t_ROM_ = −2.059, *p* = 0.050; t_std-ROM_ = −2.670, *p* = 0.013), as well as decreased ROM of the ipsilateral knee in the coronal plane (*t* = −2.148, *p* = 0.042), in the LBP group compared to the control group. For the asymptomatic controls, significantly larger ROM and ROM-std were observed in the hip and knee joints on the ipsilateral side in contrast to the contralateral side (3.287 ≤ *t* ≤ 4.500, 0.001 ≤ *p*≤ 0.006), but no significant differences were found between the two sides in the LBP group. In addition, the LBP group showed significantly lower RMS of the biceps femoris than the control group (t_RMS_ = −2.186, *p* = 0.044).

**Discussion:** Patients with LBP showed a conservative postural control pattern, characterized by reduced ROM of ipsilateral joints and diminished activation of the biceps femoris. These findings suggested the importance of voluntary postural control assessment and intervention to maximize recovery.

## 1 Introduction

Low back pain (LBP) has become a serious global public health concern (Dionne et al., 2008). Data from the Global Burden of Disease Study 2017 indicated that the worldwide point prevalence of LBP was estimated at 7.5% (Wu et al., 2020). LBP is characterized by a persistent condition of pain with a variable course, rather than unrelated episodic occurrences (Hartvigsen et al., 2018). Evidence suggested that although a portion of patients experiencing acute LBP might achieve complete relief within a few weeks, approximately two-thirds of those with acute LBP continue to endure persistent or fluctuating low to moderate pain, which can extend for up to 12 months (Heuch and Foss, 2013; Itz et al., 2013). The social burden of prolonged LBP is considerable, with reported risks of early retirement resulting in a fourfold reduction in personal income and indirect productivity loss (amounting to AU $2.9 billion) in Australia’s GDP per year (Schofield et al., 2012).

Postural control depends on continuous communication between sensory and motor systems, while LBP disrupts both sensory and motor aspects (Brumagne et al., 2019). At the stage of sensory input, patients with LBP showed diminished somatosensory perception, including lumbar tactile acuity (Adamczyk et al., 2018) and proprioceptive acuity of the lumbar spine (Lin et al., 2019), lumbopelvic complex (Korakakis et al., 2021), and lower limbs (Rosa et al., 2016). With regard to motor dysfunction, some evidence showed decreased muscle strength of hip abductor/extensors and knee extensors among LBP patients (de Sousa et al., 2019), with limited joint range of motion (ROM) in the lumbar spine (Laird et al., 2014) and hip joints (Avman et al., 2019). As a result, these sensory and motor impairments in patients with LBP may contribute to a deterioration in postural control ability.

However, the impact of LBP varies on both postural control components: involuntary (Reeves et al., 2011) and voluntary postural control (Ustinova et al., 2003). Because the human body is similar to an inverted pendulum model, the center of mass involuntarily oscillates during static standing (Reeves et al., 2011). A recent meta-analysis revealed that patients with LBP exhibited greater postural sway amplitude during static standing compared to those without pain (Park et al., 2023), suggesting impaired involuntary postural control in patients with LBP. Voluntary postural control involves perceiving and controlling the amplitude of weight shifting in self-initiated tasks (Le Mouel and Brette, 2017). Most activities of daily living, such as sit-to-stand or walking, comprise a component of voluntary postural control. From sitting to standing, the continuous contraction of the muscles of the low back and lower limbs allows for smooth and accurate voluntary postural control (Wang et al., 2023). During sitting to standing, patients with LBP demonstrated limited the ROM of lumbar spine and hip joints and prolonged transition time from sitting to standing (Sedrez et al., 2019). These changes in kinematics suggest a conservative postural control strategy in patients with LBP as they voluntarily restrict the amplitude of weight shifting to maintain postural stability in the sit-to-stand task.

In terms of gait changes, patients with LBP demonstrated reduced gait speed and shorter stride length compared to pain-free controls (Smith et al., 2022). To initiate walking, body weight should be voluntarily shifted to one side to facilitate the stride on the other. When the ipsilateral side of the lower limb transitions from the support phase to the swing phase, effective weight shifting to the contralateral side can be beneficial for enhancing the gait cycle (Alcock et al., 2013). A systematic review showed that it may be challenging for patients with LBP to maintain normal gait and stable posture consecutively, possibly due to impaired control of lateral weight shifting (Smith et al., 2022).

However, there is a paucity of evidence in patients with LBP regarding changes in the postural control pattern during lateral weight shifting. Chen et al. designed and validated a purpose-designed device, the Sway Discrimination Apparatus (SwayDA) (Chen et al., 2019). In the SwayDA test, participants are required to laterally sway to a series of preset distances and identify the differences, which provides a model for mimicking lateral weight shifting movements when initiating gait. The aim of this study was to investigate the differences in kinematic characteristics and muscle activation during voluntary lateral weight shifting between patients with LBP and asymptomatic controls without pain. It was hypothesized that patients with LBP exhibit a conservative control strategy during voluntary lateral weight shifting compared to asymptomatic controls.

## 2 Materials and methods

### 2.1 Design

This study is a case-control design between people with LBP and matched asymptomatic controls. Ethical approval of this study was received from the Human Research Ethics Committee at Swinburne University of Technology (ID number: 20225788-11032). Participants were recruited through social media platforms, and informed consent was obtained from each participant before they participated in the study. Additionally, a separate written informed consent, explicitly granting permission for the use of participants’ images, was also obtained for this study.

### 2.2 Participants

LBP is defined as pain localized between the costal margin and iliac crest without recognizable pathology (e.g., sciatica or spinal fracture) for LBP and not in the acute phase (after 4 weeks) (Knezevic et al., 2021). Participants without self-report pain in the back and leg in the past 6 months were allocated to the asymptomatic control group. Participants were excluded if they 1) were under 18, 2) had conditions or were taking medications that could affect balance, 3) had major injuries at the spine or lower limbs in the past 6 months (e.g., ankle sprain, fracture), or 4) were pregnant or within 3 months *postpartum*.

### 2.3 Instrumentation

#### 2.3.1 Clinical evaluation

A numeric rating scale (NRS) was used to rate the severity of pain from 0 (no pain) to 10 (worst pain imaginable) in people with LBP was measured by in people with LBP (Chiarotto et al., 2019). The Oswestry disability index (ODI) was used to measure LBP-related disability with a higher score indicating a greater level of disability (Chiarotto et al., 2016). In this study, the ipsilateral weight shifting towards side was the dominant side of the lower limb, which was determined by the Waterloo Footedness Questionnaire (Yang et al., 2018). The Waterloo Footedness Questionnaire typically contains 10 questions on the preferences for using either their right or left foot in various activities.

#### 2.3.2 Sway Discrimination Apparatus

**FIGURE 1:**
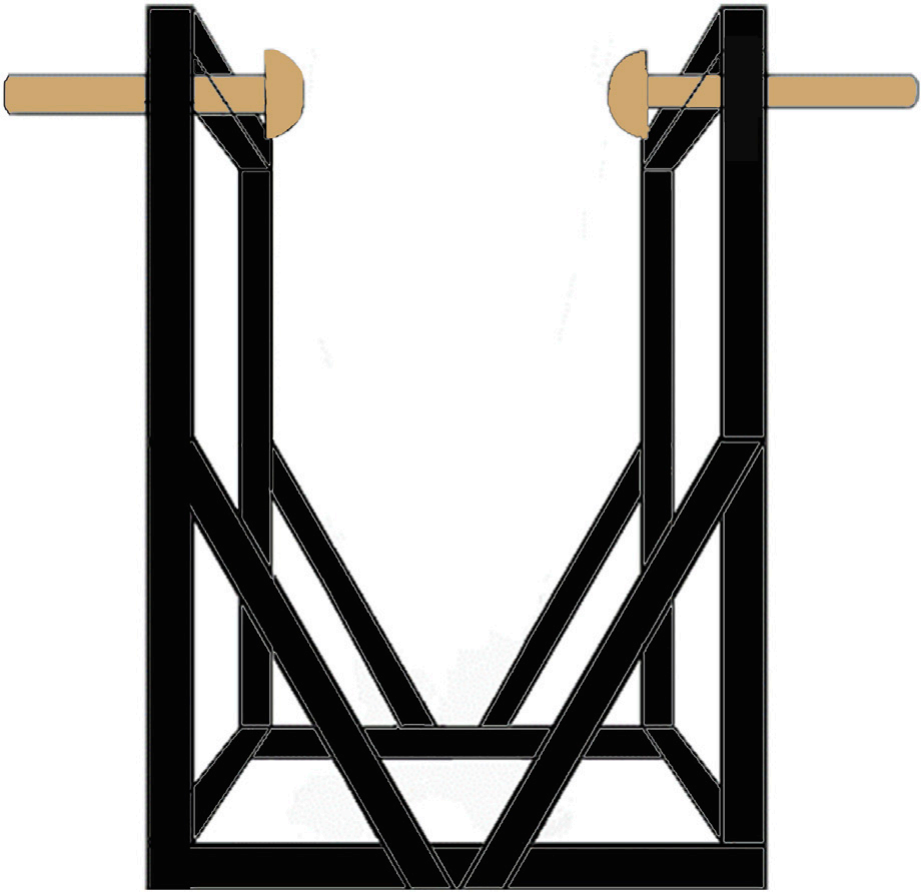
The sway discrimination apparatus for lateral wight shifting.

The SwayDA was used in this study to mimic voluntary lateral weight shifting. The structure of the SwayDA has been described in a previous study (Chen et al., 2019). Specifically, the SwayDA consists of a testing platform and two movable stops attached to both sides of the platform (Figure 1). One of the movable stops provides position information for the initial position (neutral standing at the center of the platform), while the other movable stop is set with four gradually increasing amplitudes for lateral weight shifting (4, 4.5, 5, and 5.5 cm).

#### 2.3.3 Surface electromyograph

**FIGURE 2:**
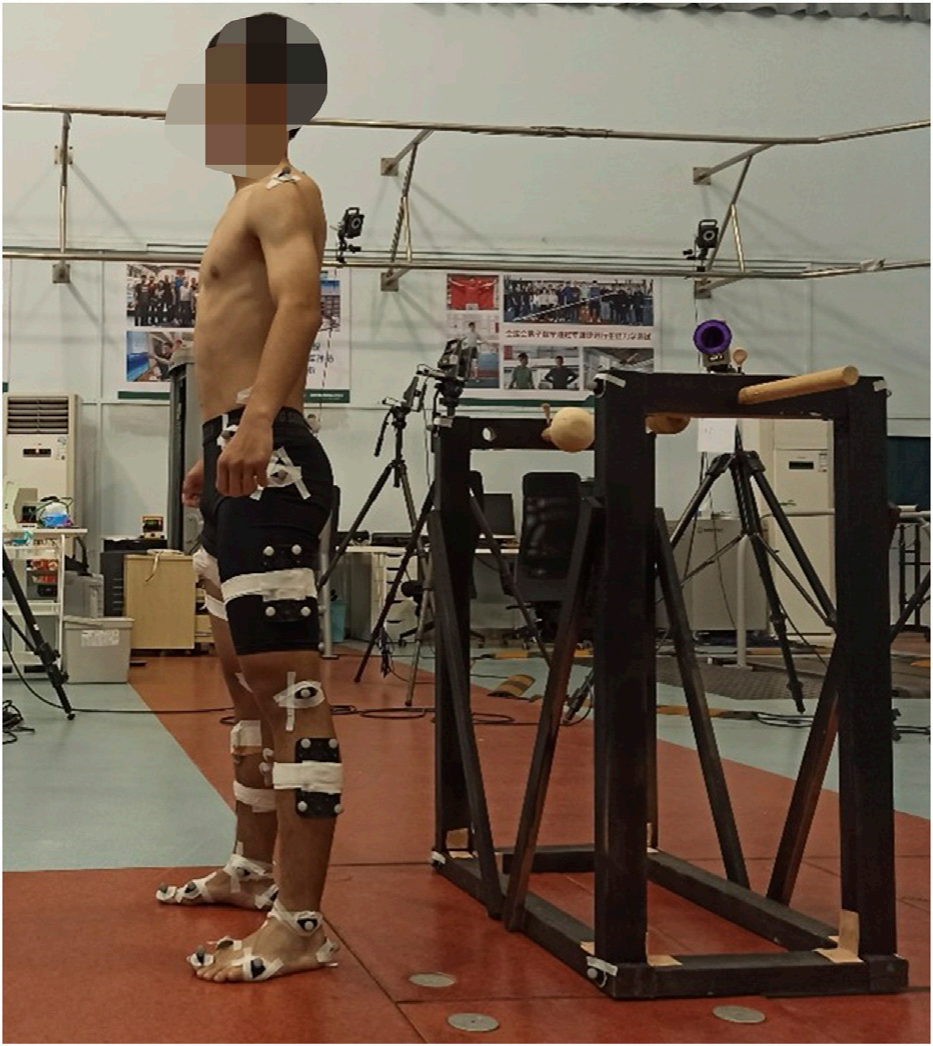
The testing environment of surface electromyography and motion capture system.

Noraxon Ultium surface electromyography (sEMG) sensor system (Noraxon USA Inc., Scottsdale, AZ, United States) was employed in this study. Signals were collected at a 2,000 Hz sampling frequency using disposable Ag-Ag Cl electrodes, with a 20 mm separation between them. The skin of each participant was shaved and cleaned with alcohol. As shown in Figure 2, the electrodes were then placed on the ipsilateral side for the following eight muscles: (1) erector spinae (longissimus thoracis pars lumborum), (2) gluteus major, (3) gluteus medius, (4) rectus femoris, (5) biceps femoris, (6) tibialis anterior, (7) gastrocnemius (medial head), and (8) peroneus longus. The placement of sensors and electrodes adhered to the recommendations of the European concerted action SENIAM (Surface Electromyography for the Non-Invasive Assessment of Muscles) standard (Hermens et al., 2000). The detailed placement of the sEMG electrodes can be seen in Supplementary Figure S1A front view and Supplementary Figure S1B back view.

#### 2.3.4 Motion capture system

An 8-camera high-speed motion capture system (Qualisys ProReflex, Qualisys Medical AB, Gothenburg, Sweden) was utilized to collect kinematic data for the trunk and lower limbs at a sampling rate of 200 Hz. Forty-two reflective markers were applied on the trunk and lower limbs, and eight on the frame of the SwayDA (Figure 2). The placement was as follows: (1) trunk: left and right acromion, spinous process of seventh cervical spine and fourth lumbar spine; (2) pelvis: anterior inferior iliac spine, iliac crest of both sides; (3) hip: left and right greater trochanter; (4) thighs and calves: 4 markers on the side of each thigh and calf. (5) knee: medial and lateral condyles of the femur of both knees; (6) ankle and foot: medial and lateral malleolus, first, and fifth metatarsal head, first toe tip, and calcaneus of both sides; (7) the frame of the SwayDA-ML: 8 markers. The detailed placement of the markers was shown in Supplementary Figure S1C front view and Supplementary Figure S1D back view.

#### 2.3.5 Data synchronization

The digital connection was established between the Qualisys Miqus Sync Unit (Qualisys Medical AB, Gothenburg, Swede), the Noraxon Ultium receiver, and the trigger button via the Bayonet Neill-Concelman cable before the start of this study. The Trigger Function was enabled when the sEMG driver was installed in Qualisys Track Manager 2020 (Qualisys Medical AB, Gothenburg, Sweden). Synchronized acquisition of sEMG data with Motion data was then activated by tapping the trigger.

### 2.4 Procedure

**FIGURE 3:**
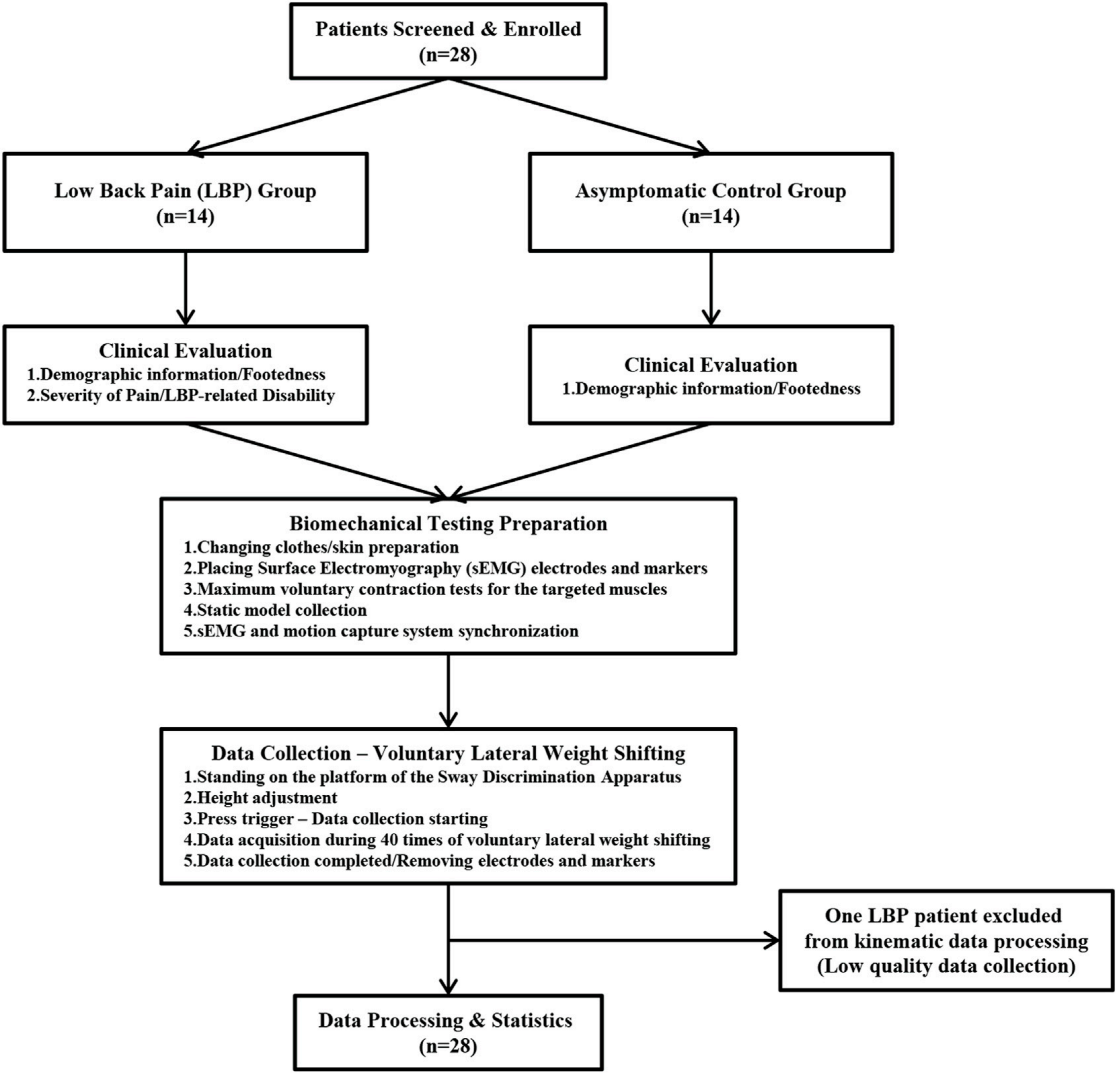
Flow chart of participants inclusion, clinical evaluation, and biomechanical data collection.

Figure 3 showed the entire testing process of this study. After the inclusion of participants, the Waterloo Footedness Questionnaire was used to determine the dominant side of the lower limbs. The severity of pain and LBP-related disability in patients with LBP were measured by NRS and ODI, respectively. Before collecting target motions, the Qualisys system was calibrated to ensure data quality. Participants were required to wear a tight non-reflective swimsuit, and the sensors, electrodes, and reflective markers were all placed in predetermined positions. Maximum voluntary contraction (MVC) of the targeted muscles was performed and recorded to normalize sEMG data with reference to previous studies (Perotto, 2011; Bernard et al., 2017). Participants were instructed to gradually contract their muscles until they reached their maximum voluntary effort within 5 s and to maintain this muscle contraction for an additional 5 s. Verbal encouragement was offered to motivate the participants during MVC testing. There were two consecutive MVC trials for each muscle, with a 1-min interval between the trials.

In the static model collection, participants stood in front of the SwayDA with their feet shoulder-width, arms on the side, palms forward, and eyes front. The accuracy and omissions of 50 markers were checked in this session. The collected static models would be used to calibrate the coordinate axis and segment coordinate systems.

Before the lateral weight shifting acquisition session, the participants stood on the platform of the SwayDA, and the movable stop was adjusted to the level of the participants’ greater trochanter. The participants were then asked to laterally shift weight to the dominant side and return to the neutral standing position after touching the moveable stop. Weight shifting was initiated from the ankles, resembling an inverted pendulum model that not only tilted the hip to the side. During the acquisition session, the four weight shifting amplitudes were randomly presented 10 times each, totaling 40 shifts. The sEMG and motion capture system were synchronously activated, recording the muscle electrical activity and kinesthetics of the trunk and lower limbs of the participants during 40 weight shifts in the SwayDA test.

### 2.5 Data processing

#### 2.5.1 Kinematic data

Raw kinematic data during the 40 times voluntary lateral weight were preconditioned and exported by Qualisys Track Manager 2020 and processed via Visual 3D Professional 2020 (CMotion Inc., Maryland, United States) to construct a rigid body model with foot, shank, thigh, pelvis, and trunk. Joint angles are defined as the differences in Eulerian angles between the two body segments that make up a joint (Tak et al., 2020). The spine, hip, knee, and ankle rotation were referenced to their proximal segment, while the pelvic segment rotation was referenced to the ground. Joint angles were computed using the Cardan rotation sequence (X-Y-Z), which is equivalent to the Joint Coordinate System. The sequence X-Y-Z represented the joint motion in the sagittal plane (flexion/extension), the coronal plane (abduction/adduction), and the transverse plane (internal/external rotation), respectively. The trajectory of each body segment was converted into real-time joint angle change for each joint by the built-in pipeline in Visual 3D. ROM was defined as the range of joint angle changes during the 40 trials in the SwayDA test, and std-ROM was the standard deviation of joint angle changes.

#### 2.5.2 Surface electromyographic data

The raw data from the sEMG sensors were processed using MATLAB R2021a (Math- Works, Inc., Natick, MA, United States). Raw sEMG signals of the 40 shifts were bandpass filtered (20–500 Hz) using a fourth-order Butterworth filter, rectified, and lowpass filtered with a cutoff frequency of 6 Hz for smoothing (Rose-Dulcina et al., 2019). The process sEMG signals were then standardized using the MVC value of the targeted muscles, and the root means square (RMS) was calculated in a pre-programmed code sequentially (unit: %MVC). The RMS denotes the square root of the mean power of the EMG signal within a specified time interval and indicates the activation level of the motor units to which the target muscles belong.

### 2.6 Statistical analysis

The significance level was set at 0.05. Normally distributed outcomes were presented as mean ± SD, while non-normally distributed results were presented as median [25th, 75th percentile]. An independent *t*-test (or Mann-Whitney *U* test for non-normally distributed outcomes) was employed in the comparison of demographic measures, kinematics (ROM and Std-ROM), and muscle activation (RMS) between the LBP and control groups. To compare the differences in kinematic measures between the ipsilateral side and contralateral side, a paired-sample *t*-test (or Wilcoxon test for non-normally distributed outcomes) was used with Bonferroni’s corrected significance level of 0.025 (Kim and Park, 2019). For t-tests, effect sizes were calculated using Cohen’s d, and effect size (r) was used to calculate the power of the nonparametric tests (Wang et al., 2022). The power of all tests was calculated by Gpower 3.1.9 (Universität Düsseldorf, North Rhine-Westphalia, Germany) (Faul et al., 2007). All statistical analyses were performed on the SPSS version 29 (IBM Corp, Seattle, United States).

## 3 Results

### 3.1 Clinical measurement

The demographic information is shown in Table 1. The mean severity of pain (±SD) in the LBP group was 3.4 ± 1.3 with pain duration 21.9 ± 16.2 months. No significant differences were found between the LBP group and control group in the demographics (all *p* > 0.05).

**TABLE 1:** Demographic characteristics in people with and without LBP (mean ± SD).

	Low back pain group	Control group
Number	14	14
Age	21.2 ± 2.9	23.9 ± 1.5
Gender (Male/Female)	5/9	7/7
Height	170.1 ± 9.6	170.5 ± 9.8
Weight	65.1 ± 14.4	63.9 ± 14.4
Numeric Rating Scale	3.4 ± 1.3	
Pain Duration (Month)	21.9 ± 16.2	
Oswestry Disability Index	9.0 ± 4.5	

### 3.2 Kinematics

The data from one participant in the LBP group was excluded due to poor motion capture quality, as there were too many missing markers that could not be rectified. The ROM of the spine, pelvis, hip, knee, and ankle joints during lateral weight shifting are demonstrated in Table 2. As shown in Supplementary Figure S2, the LBP group had significantly lower ROM of ipsilateral hip joint in the coronal and transverse planes than the control group (LBP: 6.60 [5.50, 8.42] vs. Control: 8.56 [7.40, 11.21], and LBP: 7.99 [6.61, 10.71] vs. Control: 13.87 [11.47, 17.86], respectively, both *p* < 0.05). Similarly, the ROM of the ipsilateral knee that traveled in the coronal plane was significantly lower in the LBP group than in the control group (LBP: 6.17 [4.33, 7.79] vs. Control: 9.29 [6.29, 14.02], *p* = 0.033).

**TABLE 2:** Differences in the range of motion of the spine, pelvis, and lower limb joints that traveled in sagittal, coronal, and transverse planes between the low back pain group and the control group [median (25th percentile, 75th percentile)^a^, unit: degree].

	Low back pain group (*n* = 13)	Control group (*n* = 14)	*p*	Effect size (r)	Power (1-β)
Spine—Range of Motion					
Sagittal Plane	4.17 [3.14, 5.96]	4.06 [3.52, 6.16]	0.808	−0.047	0.810
Coronal Plane	5.92 [4.24, 8.57]	5.89 [4.76, 9.85]	0.846	−0.037	0.847
Transverse Plane	5.55 [4.58, 7.64]	5.17 [4.70, 7.10]	0.734	−0.065	0.739
Pelvis—Range of Motion					
Sagittal Plane	3.05 [1.94, 4.52]	3.30 [3.21, 3.76]	0.467	−0.140	0.509
Coronal Plane	3.41 [2.42, 4.21]	3.54 [3.09, 4.67]	0.225	−0.233	0.342
Transverse Plane	5.33 [4.72, 6.59]	5.76 [4.67, 6.66]	0.734	−0.007	0.734
Ipsilateral^b^ Hip—Range of Motion					
Sagittal Plane	5.99 [3.71, 7.18]	6.79 [4.27, 12.92]	0.409	−0.159	0.466
Coronal Plane	6.60 [5.50, 8.42]	8.56 [7.40, 11.21]	**0.037** ^c^	−0.402	0.200
Transverse Plane	7.99 [6.61, 10.71]	13.87 [11.47, 17.86]	**0.009** ^c^	−0.504	0.140
Contralateral Hip—Range of Motion					
Sagittal Plane	4.24 [3.05, 6.29]	3.33 [2.92, 4.02]	0.145	0.280	0.291
Coronal Plane	6.19 [4.94, 9.38]	5.89 [4.03, 6.98]	0.174	0.262	0.310
Transverse Plane	6.84 [4.57, 9.11]	5.24 [4.27, 7.70]	0.264	0.215	0.367
Ipsilateral Knee—Range of Motion					
Sagittal Plane	9.42 [7.15, 13.65]	13.07 [5.95, 14.49]	0.627	−0.093	0.642
Coronal Plane	6.17 [4.33, 7.79]	9.29 [6.29, 14.02]	**0.033^c^**	−0.411	0.194
Transverse Plane	11.01 [8.45, 16.78]	16.80 [9.95, 25.71]	0.058	−0.364	0.223
Contralateral Knee—Range of Motion					
Sagittal Plane	5.26 [4.50, 8.72]	4.20 [3.14, 6.17]	0.073	0.345	0.238
Coronal Plane	3.51 [2.62, 4.8]	3.38 [2.47, 4.49]	0.734	0.065	0.739
Transverse Plane	7.43 [6.56, 10.87]	7.33 [4.54, 9.47]	0.332	0.187	0.412
Ipsilateral Ankle—Range of Motion					
Sagittal Plane	6.84 [3.19, 8.11]	4.30 [2.90, 9.98]	0.662	0.084	0.673
Coronal Plane	7.14 [5.51, 10.1]	5.84 [5.16, 12.93]	0.846	0.037	0.847
Contralateral Ankle—Range of Motion					
Sagittal Plane	3.85 [3.12, 5.58]	3.21 [2.57, 4.04]	0.244	0.224	0.354
Coronal Plane	6.57 [4.79, 9.56]	6.58 [5.75, 8.64]	0.846	−0.037	0.847

^a^ Mann-Whitney *U* test was used to compare the differences between the low back pain group and the control group due to non-normality.

^b^ The ipsilateral side is the weight shifting towards side (dominant side).

^c^ Significant difference between groups at 0.05 level.

As depicted in Table 3, there were no significant differences in the ROM of the hip and ankle joints between the ipsilateral side and the contralateral side in the LBP group (all *p* > 0.025). Contrary to this, Table 4 revealed that in the control group, the ROM of the hip and knee joints on the ipsilateral side was significantly larger in all three planes compared to the ROM of the contralateral side (0.001 ≤ *p* ≤ 0.006).

**TABLE 3:** The differences in the range of motion of the hip, knee, and ankle joints between the ipsilateral weight shifting towards side (dominant side) and contralateral side in the low back pain group (*n* = 13)^a^ ^,^ ^b^.

Range of motion	*p*	Effect size (r)	Power (1-β)
Hip-Sagittal Plane	0.279	0.300	0.783
Hip-Coronal Plane	0.753	0.087	0.785
Hip-Transverse Plane	0.279	0.300	0.783
Knee-Sagittal Plane	**0.023^c^**	0.630	0.936
Knee-Coronal Plane	**0.019^c^**	0.649	0.940
Knee-Transverse Plane	0.173	0.378	0.834
Ankle-Sagittal Plane	0.087	0.475	0.884
Ankle-Coronal Plane	0.807	0.068	0.823

^a^ Note: Wilcoxon test was used to compare the differences between the ipsilateral and contralateral sides due to non-normality.

^b^ Bonferroni’s corrected significance level of 0.025 due to multiple comparisons.

^c^ Significant difference at 0.025 level

**TABLE 4:** The differences in the range of motion of the hip, knee, and ankle joints between the ipsilateral weight shifting towards side (dominant side) and contralateral side in the control group (*n* = 14)^a^ ^,^ ^b^.

Range of motion	*p*	Effect size	Power (1-β)
Hip-Sagittal Plane	**0.006** ^c^	0.730	0.961
Hip-Coronal Plane	**0.001** ^c^	0.881	0.979
Hip-Transverse Plane	**0.001** ^c^	0.881	0.979
Knee-Sagittal Plane	**0.002** ^c^	0.814	0.969
Knee-Coronal Plane	**0.002** ^c^	0.847	0.981
Knee-Transverse Plane	**0.001** ^c^	0.864	0.973
Ankle-Sagittal Plane	0.048	0.528	0.913
Ankle-Coronal Plane	0.433	0.210	0.732

^a^ Wilcoxon test was used to compare the differences between the ipsilateral and contralateral sides due to non-normality.

^b^ Bonferroni’s corrected significance level of 0.025 due to multiple comparisons.

^c^ Significant difference at 0.025 level.

The Std-ROM reflects the variation of ROM during the voluntary weight shifting, as shown in Supplementary Table S1. Compared with the control group, the LBP group showed significantly smaller Std-ROM of the ipsilateral hip joint in the coronal and transverse plane (LBP: 1.19 [0.78, 1.54] vs. Control: 1.76 [1.19, 1.98], and LBP: 1.17 [0.95, 1.52] vs. Control: 2.16 [1.54, 3.09]), respectively, both *p* < 0.05). Supplementary Tables S2, S3 compared the differences in Std-ROM of multiple joints of the lower limb between the ipsilateral side and contralateral side in the LBP group and the control group, respectively, where no significant differences in Std-ROM of the hip, knee, and ankle joints in the LBP group (Supplementary Table S2, all *p* > 0.025). In the control group (Supplementary Table S3), however, the Std-ROM of the hip and knee joints on the ipsilateral side was significantly larger than on the contralateral side in sagittal, coronal, and horizontal planes (0.002 ≤ *p*≤ 0.009).

### 3.3 Surface electromyography

The RMS of the spine and leg muscles is shown in Table 5. The RMS of the biceps femoris in the LBP group was significantly lower (LBP: 0.90 [0.63, 1.89] vs. Control: 1.90 [1.45, 4.53], *p* = 0.043). However, the RMS of the other muscles did not show significant differences between groups throughout the 40 voluntary weight shifts in the SwayDA test (Supplementary Figure S3).

**TABLE 5:** The differences in the root mean square of the targeted eight muscles of the dominant side during voluntary lateral weight shifting (median [25th percentile, 75th percentile], unit: %MVC).

Root mean square	Low back pain group (*n* = 14)	Control group (*n* = 14)	*p*	Effect size (r)	Power (1-β)
Gastrocnemius	2.70 [1.32, 3.61]	2.95 [0.82, 5.28]	0.818	−0.043	0.820
Tibialis Anterior	1.30 [0.74, 3.19]	2.76 [0.66, 4.94]	0.435	−0.148	0.485
Peroneus Longus	2.76 [1.51, 3.70]	3.51 [2.33, 5.41]	0.232	−0.226	0.347
Biceps Femoris	0.90 [0.63, 1.89]	1.90 [1.45, 4.53]	**0.043** ^a^	−0.382	0.207
Rectus Femoris	0.54 [0.34, 0.92]	0.44 [0.19, 1.85]	0.854	−0.035	0.855
Gluteus Maximus	1.42 [0.80, 3.05]	1.64 [0.50, 4.47]	0.818	−0.043	0.820
Gluteus Medius	3.32 [1.66, 6.26]	3.44 [1.75, 5.67]	0.963	−0.009	0.963
Erector Spinae	4.43 [1.94, 7.20]	5.52 [3.80, 7.40]	0.383	−0.165	0.447

^a^ Significant difference between groups at 0.05 level.

## 4 Discussion

This study explored kinematic parameters and muscle electrical activity at the lumbar spine, pelvis, and lower limbs of patients with LBP and asymptomatic controls during voluntary lateral weight shifting. The results showed that patients with LBP showed a conservative postural control strategy characterized by limiting the ROM of the hip and knee at the ipsilateral side compared to the control group when voluntarily shifting weight. The asymptomatic control group demonstrated notable lateralization in controlling voluntary weight shift, as evidenced by significantly larger ROM and ROM-std observed in the lower limb on the ipsilateral side in contrast to the contralateral side. Conversely, movement in both lower limbs exhibited no significant differences in all directions of joint ROM and ROM-std in the LBP group.

Most activities of daily living are voluntary postural control tasks, which require individuals to voluntarily shift their weight to facilitate task performance and maintain postural stability. Compared to the weight shifting in the sagittal plane, the lateral weight shifting model used in this study is more challenging and energy-consuming (Reeves et al., 2011). In a gait cycle, lateral weight shifting occurs before initiating a step. Sufficient weight shifting can help maintain a stable posture on a single-leg basis during the support phase, allowing the other lower limb to be fully cleared for a normal stride and pace. Due to the importance of voluntary lateral weight shifting in activities of daily living, this study explores changes in kinematic characteristics and muscle activation in the lateral weight shifting model in patients with LBP.

In this study, the LBP group showed a significant reduction in the ROM of the ipsilateral hip joint in the transverse plane and the ROM of the ipsilateral knee joint in the coronal plane. These findings align with our hypothesis that patients with LBP exhibit a conservative postural control pattern. The influence of LBP on joint kinematic parameters could be explained by employing conservative postural control strategies that aim to attain postural stability via promoting stiffness in the spine and lower limbs (Zafereo et al., 2015). There is currently no consensus regarding the changes in knee joint kinematic parameters in patients with LBP, since the knee is not directly connected to the lumbar spine, and the function of the knee joint varies significantly across different activities. During running, patients with LBP use a knee stiffness strategy to stabilize the knee joint (Hamill et al., 2009). In the task of landing from a 30 cm height box, no significant differences were observed in the kinematics of the knee joint between patients with LBP and asymptomatic controls (Sutherlin et al., 2020). In this study, the knee joint ROM of the ipsilateral side was reduced in the lateral weight shifting model, suggesting that the knee joint exhibits a conservative pattern consistent with the hip joint.

Voluntary lateral weight shifting is an asymmetric movement model. The control group showed a lateralized postural control pattern, wherein the ipsilateral side was primarily involved in weight shifting. Contrary to this, there was no discrepancy in the kinematic parameters between the ipsilateral side and the contralateral side in the LBP group. In tasks involving postural control in a standing position, proprioception from the lower limbs serves as a crucial source of somatosensory perception (Goodworth et al., 2014). Evidence showed a decrease in lumbar-pelvic proprioception (Korakakis et al., 2021) as well as knee proprioception (Ranjbar et al., 2023) among patients with LBP. When the proprioceptive acuity of the lower limbs and lumbar spine diminishes, the somatosensory cortex lacks adequate afferent signals to guide voluntary weight shifting. In the control group, the kinematic pattern revealed that the lower limb on the ipsilateral side dominates the process. In patients with LBP, a general reduction in proprioception may lead to a conservative postural control pattern aimed at decreasing the potential risk of postural instability during voluntary weight shifting.

The biceps femoris is located on the posterior lateral side of the thigh and is the main component of the hamstring, involving the movement of the hip, knee, and pelvis. The biceps femoris is crucial for knee functional movement and stability (Tubbs et al., 2006). The biceps femoris is affected in approximately 84% of hamstring injuries (Ekstrand et al., 2012; Ekstrand et al., 2016), and the recurrent injury rate of the biceps femoris is significantly higher than that of the semitendinosus and semimembranosus (Ekstrand et al., 2016). The activation level of the biceps femoris significantly decreased in patients with LBP compared to the asymptomatic control group in this study. The low activation of the biceps femoris muscle may be due to reduced hip and knee activity (Ertelt, 2014) in the LBP group. A study showed that patients with LBP had hamstring shortening caused by extended periods of hip and knee joint inactivity, such as a sedentary lifestyle (Shamsi et al., 2020), which affects the activation of biceps femoris. In addition to mechanical factors, sensorimotor feedback plays an important role in influencing the activation of biceps femoris (Ertelt and Gronwald, 2017), as decreased lower limb proprioception and sensory organization of somatosensory perception were found in the patients with LBP (Meier et al., 2019). As a result, the diminished activation of the biceps femoris may worsen the severity of pain in the lower back, transmitted through the lumbo-pelvic-hip complex (Redmond et al., 2014; Ertelt and Gronwald, 2017). In addition, gait may be affected by decreased activation of the biceps femoris muscle, leading to limited hip and knee movement during walking.

### 4.1 Strength and limitations

In methodology, this study used biomechanical equipment to quantitatively evaluate muscle activity and kinematic characteristics during voluntary lateral weight shifting. However, the participants included in the LBP group had mild to moderate pain (NRS ranged from 2 to 6). The participants included in this study were young patients with LBP (age 22.5 ± 2.6). Postural control patterns in patients with LBP may change due to aging, where older adults with LBP have shown reduced muscle strength in back extensors (Kienbacher et al., 2016), poorer postural instability (da Silva et al., 2016), and lower core muscle activation (da Silva et al., 2019), compared with young patients with LBP. This suggests that the kinematic characteristics and muscle electrical activity obtained herein may not be generalizable to patients with LBP of all ages. Future research should explore the changes in somatosensory perception and voluntary postural control across varying levels of pain and distinct subgroups (including sciatica) within the LBP population. Even though there were significant differences in the ROM of the ipsilateral hip and knee and the RMS of the biceps femoris between the LBP group and the control group, the power of these statistical tests was low. The low power may be related to the small sample size of this study, suggesting the potential for type II errors in the between-group differences when interpreting the results. Further research with a larger sample size is required to confirm the robustness of this finding. In addition, the findings cannot establish a causal relationship between pain and changes in voluntary postural control patterns. Prospective cohort studies may be necessary to observe the influence of pain on voluntary lateral weight shifting and to explore potential intervention methods for individuals with LBP.

### 4.2 Clinical implications

This study suggests that the postural control pattern of individuals with LBP changed, marked by the adoption of conservative control strategies and low activation of the biceps femoris during voluntary lateral weight shifting. Therefore, clinical practitioners should monitor the changes in the voluntary postural control pattern, especially in hip and knee joints at an early stage, and regularly screen the performance of tasks requiring voluntary postural control, such as walking or climbing stairs. Interventions targeting voluntary postural control may also be important for LBP patients.

## 5 Conclusion

Postural control patterns are significantly changed in the LBP group when compared to the control group during voluntary lateral weight shifting. Individuals with LBP use conservative postural control strategies during voluntary shifting weight tasks. Lower activation of biceps femoris in the LBP group suggests diminished motor control of the hip and knee joints during voluntary lateral weight shifting, which is consistent with the kinematic characteristics observed in patients with LBP. In clinical practice, it is essential to recognize the significance of assessment of voluntary postural control in patients with LBP in the early stages as this may inform targeted interventions for optimal patient outcomes. Future research should explore the association between pain and voluntary postural control adjustment across different levels of pain within the LBP population to optimize recovery.

## Data Availability

The raw data supporting the conclusion of this article will be made available by the authors, without undue reservation.
