# Supplementary material for: Kinematic changes of the trunk and lower limbs during voluntary lateral sway postural control in adults with low back pain
Source: Front Bioeng Biotechnol. 2024 Feb 27;12:1351913. doi: 10.3389/fbioe.2024.1351913 (PMC10927732; doi:10.3389/fbioe.2024.1351913)
Supplement: Supplementary file 1 [file DataSheet2.PDF]

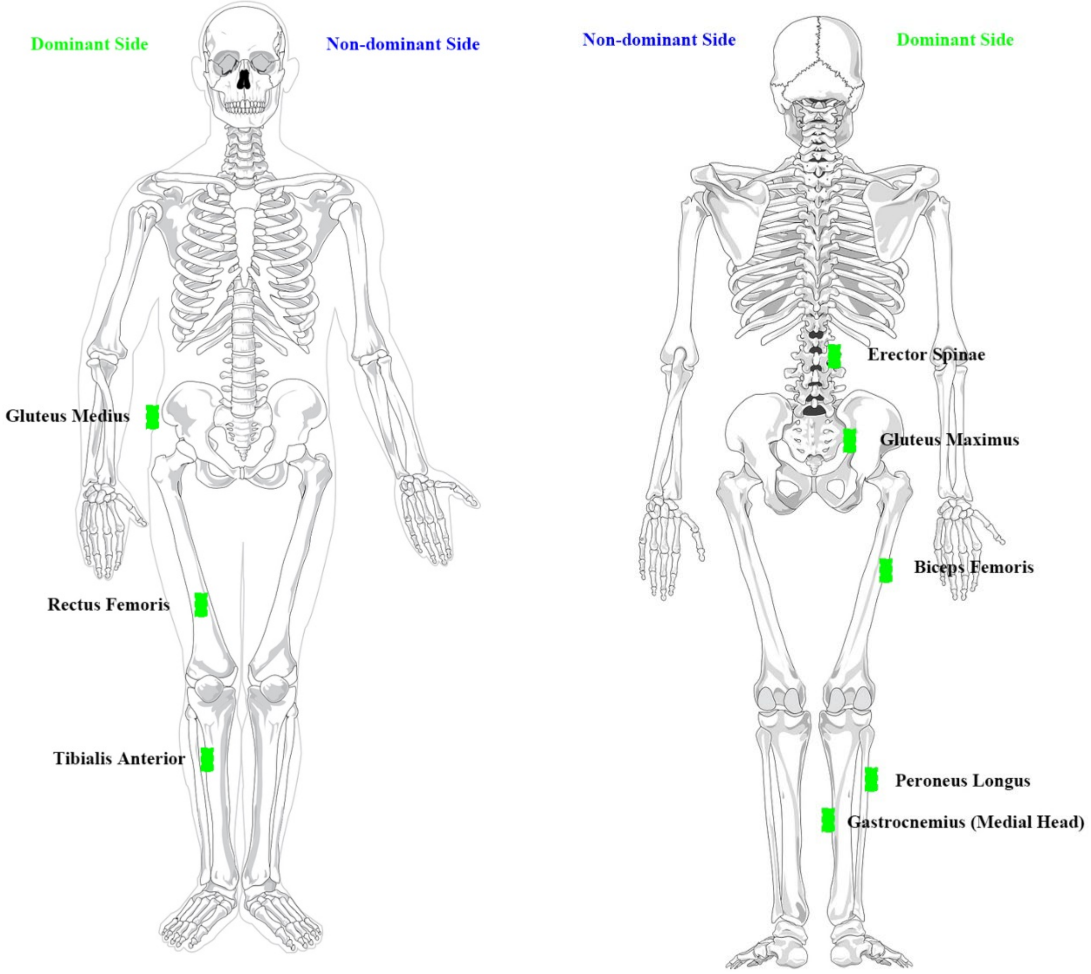

Dominant Side
Non-dominant Side
Gluteus Medius
Rectus Femoris
Tibialis Anterior
Non-dominant Side
Dominant Side
Erector Spinae
Gluteus Maximus
Biceps Femoris
Peroneus Longus
Gastrocnemius (Medial Head)

(a) (b)

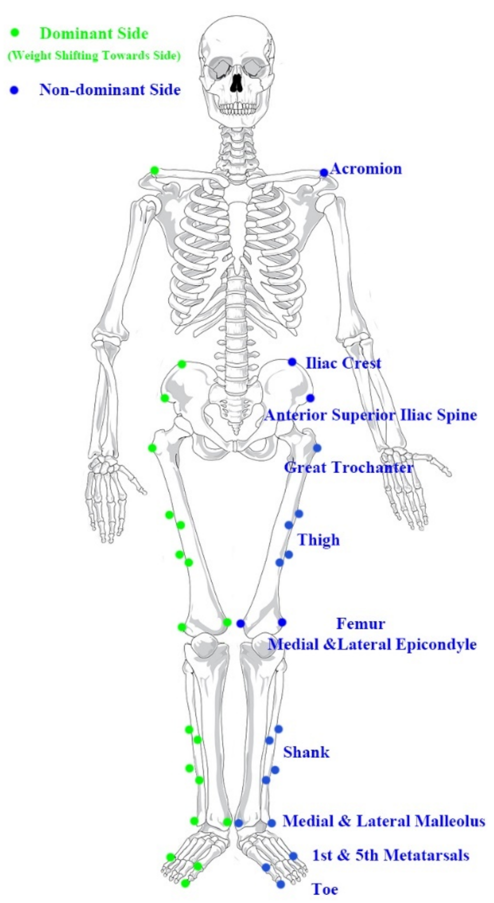

(c)

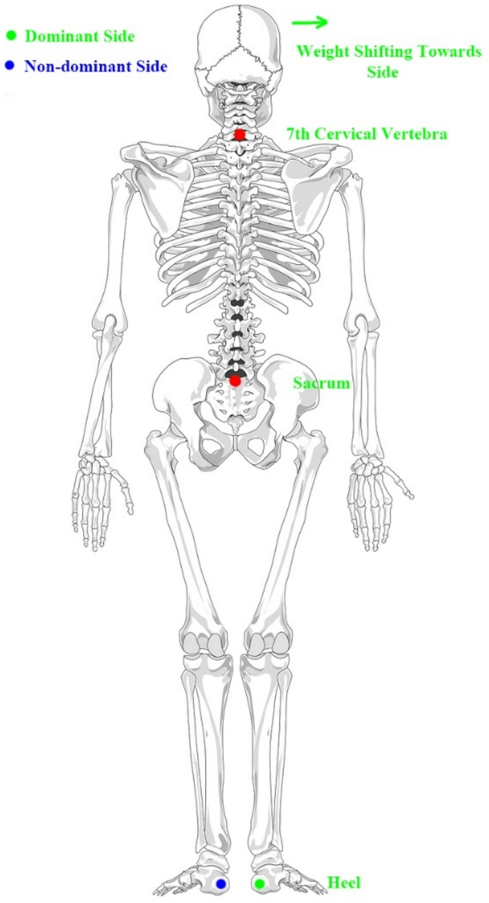

(d)

Figure S1 Markers and surface electromyography (sEMG) electrode placement. (a) sEMG electrodes placement - front view; (b) sEMG electrodes placement - back view; (c) Markers placement - front view; (d) Markers placement - back view, green arrow points to weight shifting toward side.

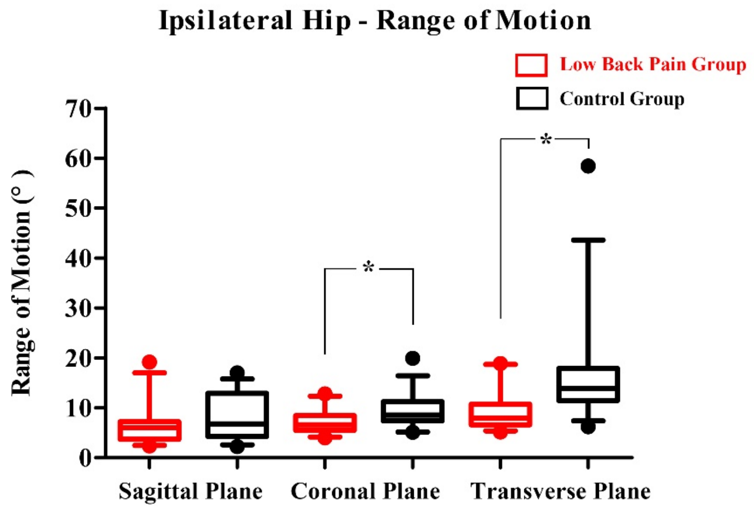

Figure S2 Differences in the hip range of motion at ipsilateral voluntary weight shift side between the low back pain group and the control group. * indicates $p<0.05$

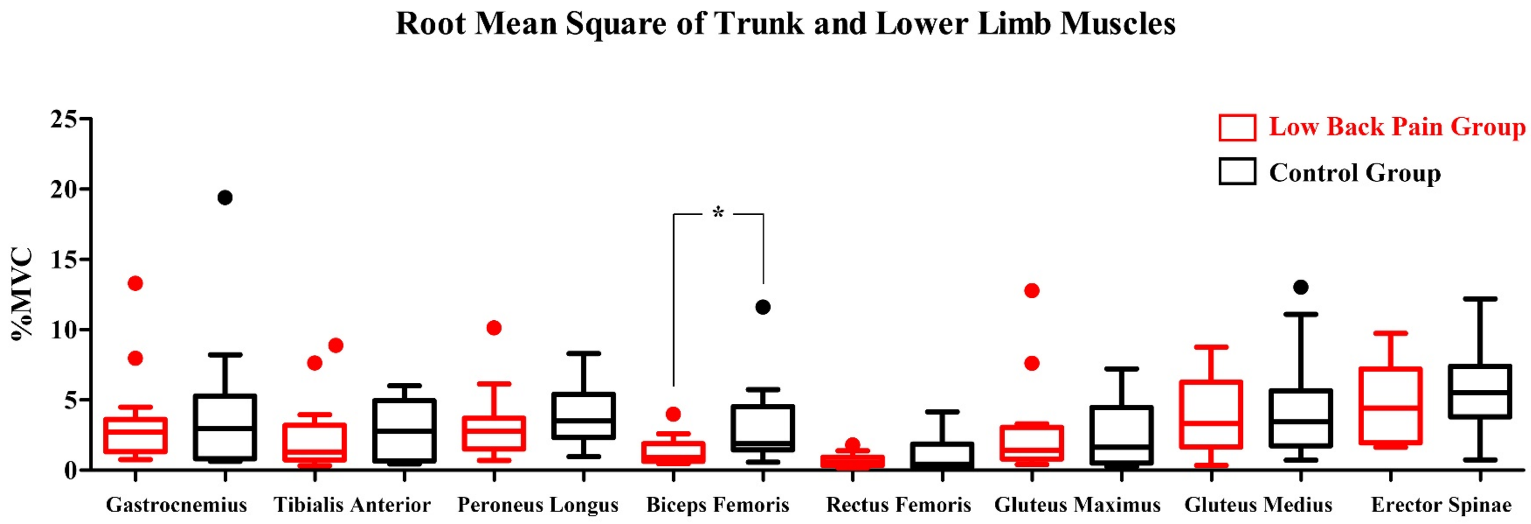

Figure S3 Differences in the root mean square of the trunk and lower limb muscles between the low back pain group and the control group. * indicates p<0.05
