# Supplementary material for: Kinematic changes of the trunk and lower limbs during voluntary lateral sway postural control in adults with low back pain
Source: Front Bioeng Biotechnol. 2024 Feb 27;12:1351913. doi: 10.3389/fbioe.2024.1351913 (PMC10927732; doi:10.3389/fbioe.2024.1351913)
Supplement: Supplementary file 2 [file DataSheet1.pdf]

Table S1 Differences in the standard deviation of range of motion of spine, pelvis, and lower limb joints between the low back pain group and the control group (median [25th percentile, 75th percentile] [a], unit: degree)

	Low Back Pain Group (n=13)	Control Group (n=14)	p	Effect Size (r)	Power (1-β)
Spine - Standard Deviation of Range of Motion					
Sagittal Plane	0.67 [0.54, 0.85]	0.72 [0.64, 1.19]	0.244	-0.224	0.354
Coronal Plane	0.97 [0.63, 1.75]	1.06 [0.81, 1.69]	0.593	-0.103	0.612
Transverse Plane	0.77 [0.60, 1.04]	0.84 [0.70, 1.13]	0.497	-0.131	0.533
Pelvis - Standard Deviation of Range of Motion					
Sagittal Plane	0.44 [0.31, 0.70]	0.59 [0.42, 0.67]	0.332	-0.187	0.412
Coronal Plane	0.54 [0.37, 0.72]	0.62 [0.47, 0.75]	0.332	-0.187	0.412
Transverse Plane	0.94 [0.83, 1.28]	0.90 [0.69, 1.11]	0.244	0.224	0.354
Ipsilateral [b] Hip - Standard Deviation of Range of Motion					
Sagittal Plane	0.79 [0.43, 1.04]	1.02 [0.73, 1.78]	0.120	-0.299	0.274
Coronal Plane	1.19 [0.78, 1.54]	1.76 [1.19, 1.98]	**0.020***	-0.448	0.171
Transverse Plane	1.17 [0.95, 1.52]	2.16 [1.54, 3.09]	**0.003***	-0.570	0.104
Contralateral Hip - Standard Deviation of Range of Motion					
Sagittal Plane	0.63 [0.51, 0.97]	0.54 [0.48, 0.62]	0.145	0.280	0.291
Coronal Plane	1.12 [0.90, 1.56]	1.10 [0.74, 1.60]	0.528	0.121	0.557
Transverse Plane	1.07 [0.82, 1.33]	0.78 [0.67, 1.41]	0.357	0.177	0.428
Ipsilateral Knee - Standard Deviation of Range of Motion					
Sagittal Plane	1.15 [0.84, 1.46]	1.29 [0.90, 2.19]	0.308	-0.196	0.395
Coronal Plane	0.78 [0.56, 1.33]	1.14 [0.71, 2.33]	0.207	-0.243	0.331
Transverse Plane	1.71 [1.33, 2.47]	2.16 [1.66, 4.30]	0.099	-0.318	0.260
Contralateral Knee - Standard Deviation of Range of Motion					
Sagittal Plane	0.89 [0.69, 1.20]	0.65 [0.41, 0.80]	**0.020***	0.448	0.171
Coronal Plane	0.56 [0.44, 0.73]	0.52 [0.36, 0.69]	0.808	0.047	0.810
Transverse Plane	1.08 [0.89, 1.35]	1.08 [0.68, 1.48]	0.662	0.084	0.673
Ipsilateral Ankle - Standard Deviation of Range of Motion					
Sagittal Plane	0.66 [0.44, 1.04]	0.67 [0.48, 1.45]	0.698	-0.075	0.706
Coronal Plane	1.16 [0.97, 1.67]	1.38 [1.03, 1.66]	0.332	-0.187	0.412
Contralateral Ankle - Standard Deviation of Range of Motion					
Sagittal Plane	0.52 [0.40, 0.73]	0.47 [0.40, 0.62]	0.528	0.121	0.557
Coronal Plane	1.30 [1.13, 1.77]	1.50 [1.15, 1.67]	0.593	-0.103	0.612

Note:
a. Mann-Whitney U test was used to compare the differences between the low back pain group and the control group due to non-normality.
b. The ipsilateral side is the weight shifting towards side (dominant side).
*: Significant difference between groups at 0.05 level

Table S2 The differences in the standard deviation of range of motion of the hip, knee, and ankle joints between the ipsilateral side and contralateral side in the low back pain group (n=13) [a, b]

Variation	p	Effect Size	Power (1-β)
Hip-Sagittal Plane	0.422	0.223	0.735
Hip-Coronal Plane	0.463	-0.204	0.726
Hip-Transverse Plane	0.221	0.339	0.809
Knee-Sagittal Plane	0.249	0.320	0.797
Knee-Coronal Plane	0.046	0.552	0.913
Knee-Transverse Plane	0.133	0.417	0.856
Ankle-Sagittal Plane	0.173	0.378	0.834
Ankle-Coronal Plane	0.463	-0.204	0.726

Note:

a. Wilcoxon test was used to compare the differences between the ipsilateral and contralateral sides due to non-normality.

b. Bonferroni's corrected significance level of 0.025 due to multiple comparisons.

Significant difference at 0.025 level

Table S3 The differences in the standard deviation of the range of motion of the hip, knee, and ankle joints between the ipsilateral side and contralateral side in the control group (n=14) [a, b]

Variation	p	Effect Size	Power (1-β)
Hip-Sagittal Plane	**0.003**#	0.797	0.972
Hip-Coronal Plane	**0.009**#	0.696	0.956
Hip-Transverse Plane	**0.002**#	0.847	0.981
Knee-Sagittal Plane	**0.002**#	0.830	0.976
Knee-Coronal Plane	**0.004**#	0.780	0.972
Knee-Transverse Plane	**0.004**#	0.780	0.972
Ankle-Sagittal Plane	0.074	0.478	0.894
Ankle-Coronal Plane	0.397	-0.226	0.740

Note:

a. Wilcoxon test was used to compare the differences between the ipsilateral and contralateral sides due to non-normality.

b. Bonferroni's corrected significance level of 0.025 due to multiple comparisons.

Significant difference at 0.025 level
